# Protein Digestibility of Cereal Products

**DOI:** 10.3390/foods8060199

**Published:** 2019-06-08

**Authors:** Iris Joye

**Affiliations:** Department of Food Science, University of Guelph, Guelph, ON N1G 2W1, Canada; ijoye@uoguelph.ca; Tel.: +1-519-824-4120 (ext. 52470)

**Keywords:** protein digestibility, cereals, pseudocereals, processing, antinutritional factors

## Abstract

Protein digestibility is currently a hot research topic and is of big interest to the food industry. Different scoring methods have been developed to describe protein quality. Cereal protein scores are typically low due to a suboptimal amino acid profile and low protein digestibility. Protein digestibility is a result of both external and internal factors. Examples of external factors are physical inaccessibility due to entrapment in e.g., intact cell structures and the presence of antinutritional factors. The main internal factors are the amino acid sequence of the proteins and protein folding and crosslinking. Processing of food is generally designed to increase the overall digestibility through affecting these external and internal factors. However, with proteins, processing may eventually also lead to a decrease in digestibility. In this review, protein digestion and digestibility are discussed with emphasis on the proteins of (pseudo)cereals.

## 1. Introduction

Proteins are abundantly present in all living cells, and as such are a vital part of our diet. In order to be usable to the human body after ingestion, dietary proteins have to be hydrolysed into their basic building blocks, i.e., amino acids or small peptides. Hereto, the human gastrointestinal tract possesses an assortment of protein hydrolysing enzymes or peptidases. The physiological roles of proteins are very diverse and range from functioning as pure nitrogen storage molecules over delivering structure building capacity to catalysing a myriad of reactions as metabolically active enzymes or serving as transporter vehicles for poorly soluble or physiologically unstable components [1]. Proteins that have a storage function are usually higher in alkaline amino acids (i.e., arginine, lysine and histidine) as these are more nitrogen-rich.

It is widely accepted that nine out of the twenty naturally occurring amino acids are essential or indispensable amino acids. These amino acids, i.e., histidine, isoleucine, leucine, lysine, methionine, phenylalanine, threonine, tryptophan, and valine cannot be synthesised by the animal organism out of materials ordinarily available to the cells at a speed commensurate with the demands for normal growth [2] and as such, need to be part of a healthy balanced diet. However, the list of indispensable amino acids has been subject to some criticism [2]. Furthermore, Food and Agriculture Organization (FAO) recommends approaching dietary amino acids as individual nutrients. As such, wherever possible, data for digestible or bioavailable amino acids should be given in food tables on an individual amino acid basis [3]. Amino acid profiles may be determined using chromatographic techniques, either relying on ion exchange or polarity differences between the amino acids [4].

In their native state, proteins are typically folded in a specific conformation driven by the unique amino acid composition of the protein. This tightly folded conformation is hampering protein digestibility. In addition, proteins often occur in supramolecular structures such as protein bodies [5,6] and/or are physically entrapped in cellular structures [7]. This supramolecular structuring further restricts the access of the hydrolytic enzymes to their substrates.

The processing of food serves several purposes, such as ensuring food safety, prolonging shelf life and increasing digestibility. In conditions where there are restrictions on the amount of food that can be digested because of, for example, shortage, easy and virtually complete digestion and absorption of food nutrients is of paramount importance. To illustrate the role of ingredient choice and processing in determining food digestibility, the digestibility of proteins in diets from developing and developed countries can be compared. Traditional diets in developing countries usually have a protein digestibility varying between 54 and 78%, while in developed countries like those diets found in North America, protein digestibility is closer to or even exceeds 90% [8]. Reasons for the lower digestibility of food in developing countries is the use of less refined ingredients and less extensive processing. Food processing triggers (usually destructive) alterations in molecular and supramolecular structures to allow digestive enzymes to gain easier access to the nutrients/biopolymers, hence, improving food digestibility. In a protein context, food processing usually leads to protein unfolding. Indeed, heating, exposure to strong acid and/or alkaline conditions, and the presence of organic molecules and emulsifiers usually leads to conformational changes and, if severe enough, even to irreversible loss of protein folding or molecular scission. Denaturation opens up the protein structure, making it more accessible for hydrolytic enzymes, and, hence, increases protein digestibility. At the same time, however, protein denaturation also exposes hydrophobic patches of the proteins that are otherwise shielded from the aqueous environment. The hydrophobic effect, parallel with other interactions that are suddenly enabled due to the conformational changes, will eventually lead to protein aggregation and decreased digestibility [9].

In this review, a general description of quality scoring of protein foods and protein digestion is given before digging deeper into digestibility of proteins in cereals, pseudocereals and their products. After assessing the effect of processing on the digestibility of ((pseudo)cereal) proteins, the role of indigestible peptides in celiac disease is discussed before concluding with a few future research priorities.

## 2. Protein Foods

Protein foods are characterized by high levels of high-quality proteins. Typical protein foods are meat, bean and dairy products. Each of these have an amino acid profile that is rich in amino acids that are considered essential/indispensable [2]. Animal-derived products generally provide the essential amino acids in the ratios needed to sustain growth and metabolic processes in the human body, while plant-based protein sources typically have suboptimal levels and ratios of the essential amino acids. However, the quality of protein should not only consider the amino acid composition, but also the digestibility and the absorption of the produced hydrolysis products in the human gastrointestinal tract. It is, for example, possible that a protein has a very good amino acid profile, but cannot be well digested and/or absorbed. For policy makers, to make recommendations on protein requirements, both factors, i.e., amino acid composition and digestibility, should be considered. The well-established substantial difference in the nutritional value of proteins from different sources [10] has been captured in a range of protein quality scoring methods. One of the most popular scoring methods is the Protein Digestibility Corrected Amino Acid Score (PDCAAS) [10,11]. PDCAAS is calculated as:

$$PDCAAS=\frac{mg\;of\;limiting\;amino\;acid\;in\;1\;g\;of\;test\;protein}{mg\;of\;same\;amino\;acid\;in\;1\;g\;of\;reference\;protein}\times fecal\;true\;digestibility$$

Milk, containing adequate levels and ratios of all of the nine essential amino acids and having a high protein digestibility has been given the maximum PDCAAS of 1.0 [12]. Eggs are another protein food that was given the maximum PDCAAS. Soy and beef have values of 0.91 and 0.92, respectively, while wheat, deficient in essential amino acids such as lysine, has a PDCAAS of only 0.42. Most cereal proteins are indeed considered incomplete protein sources due to their inferior amino acid profile relative to animal-derived products [13,14]. Sorghum has a somewhat better amino acid profile, but sorghum proteins are poorly digestible, limiting the bioavailability of its amino acids [6]. PDCAAS as low as 0.20 have been reported for sorghum [15]. A more recent protein quality score is the digestible indispensable amino acid score (DIAAS), that compares the content of all digestible essential amino acids in a protein to the level of these digestible amino acids in a reference protein. The reference protein has an indispensable amino acid sequence that is similar to the profile required by a 0.5 to 3 years old child. This newer score is considered superior to PDCAAS as it utilizes the true ileal digestibility instead of the fecal digestibility of the proteins. Amino acids, peptides and proteins that are not absorbed in the small intestine end up in the colon where they are metabolized by the microbiota. Furthermore, the truncation of PDCAAS to 1.0 means that important information on highly nutritional proteins is discarded. DIAAS is calculated as follows:

$$DIAAS=100\;\times \;\frac{mg\;of\;digestible\;dietary\;indispensable\;AA\;in\;1\;g\;dietary\;protein}{mg\;of\;the\;same\;dietary\;indispensable\;AA\;in\;1\;g\;reference\;protein}$$

The indispensable amino acid in the DIAAS equation is the amino acid that has the lowest reference ratio [16]. DIAAS values for a variety of (cooked) cereals and pseudocereals (brown rice (DIAAS is 42, first limiting amino acid is lysine), polished rice (37, lysine), buckwheat (68, sulfur containing amino acids), oats (43, lysine), proso millet (7, lysine), foxtail millet (10, lysine), tartary buckwheat (47, sulfur containing amino acids) and wholewheat (20, lysine)) show that none of the cereals can be considered to be a good protein source [16]. To put these values in perspective, whole milk, hard-boiled eggs and chicken breast have DIAAS scores of 114, 113 and 108, respectively [17]. For a more extensive review on the applicability and value of the DIAAS score, the reader is kindly referred to the review by Marinangeli and House [17].

## 3. Protein Digestion

During digestion, proteins are hydrolyzed into (small) peptides and eventually amino acids that can then be readily assimilated by the human body. Enzymes taking part in this process are called peptidases. Human peptidases are found in the stomach, pancreas and small intestine. After hydrolysis, the small peptides and amino acids should be rapidly and efficiently absorbed by the enterocytes in the small intestine. The absorption of peptides in the human intestine has been elaborately reviewed by Lundquist and Artursson [18] and falls outside the scope of this manuscript.

In the Nutrition Facts table published by Health Canada [19], there are no % daily values listed for proteins as most Canadians get enough protein through their diet and dietary protein deficiency is, hence, not considered a health concern in Canada. Furthermore, the assimilation of dietary proteins is believed to be highly efficient [20]. Research has shown that amino acids occur in blood about 10 to 20 minutes after ingestion of single amino acids or isolated proteins. For intact proteins embedded in food products, such as those consumed as part of beef, eggs or dairy products, it can require over 2 hours for the amino acids to occur in blood [3]. For some specific Western subpopulations, however, such as older adults, the enrichment of food products with proteins may be desirable to maintain muscle mass and functioning [21,22,23,24].

Generally, three different phases of protein digestion can be distinguished throughout the gastrointestinal tract [20]:

- Stomach phase: the main protein hydrolyzing activity in the stomach stems from two different pepsin types that are activated by the acid pH conditions. Initial hydrolysis products are fairly large polypeptides, a few smaller peptides and even some free amino acids. It was found, however, that this protein digestion phase is not crucial as people that had their stomach removed can still digest and assimilate protein provided through the diet.

- Pancreatic phase: this much more crucial phase in protein digestion makes use of a mixture of proteolytic enzymes produced by the pancreas, such as trypsin, chymotrypsin, elastase and carboxypeptidase A and B. The resulting hydrolysis products are a heterogenous mixture of small oligopeptides and free amino acids. At this phase, the pH has been increased, hence, inactivating the gastric peptidases. Trypsin, chymotrypsin and elastase are endopeptidases, cleaving peptide bonds in the middle of the amino acid chain. Each of these enzymes are recognizing very specific amino acids along the primary structure of their substrates. Carboxypeptidases, on the other hand, are exopeptidases that remove single amino acids from the C-terminal end of their substrates [25,26]. The catalytic complementarity of these five major pancreatic peptidases ensures proper hydrolysis of the proteins to free amino acids and oligopeptides.

- Small intestine phase: in the brush border membrane of the small intestine, an aminopeptidase N is present that hydrolyzes short oligopeptides by sequentially removing N-terminal amino acids. The enzymes in this intestinal brush border show a high specificity for proline-containing peptides. Pancreatic enzymes indeed are not able to hydrolyze peptide bonds that involve proline. Apart from these aminopeptidases, several metallo-endopeptidases are found that can completely hydrolyze proteins to their constituting amino acids. The jejunum, the middle part of the small intestine, is the hot spot for absorption for amino acids. Research also indicated that the jejunal phase, with its brush border peptidases, should not be overlooked when designing an in vitro digestion process to accurately determine the bioaccessibility and bioavailability of dietary peptides [27]. This latter part was concluded after feeding a variety of milk protein mixtures to two different in vitro procedures. The peptides arising from these models were subsequently fed into a system that contained these jejunal brush border peptidases. After the in vitro methods, the peptides and achieved degree of hydrolysis differed to some extent. After the brush border peptidase treatment, the differences in hydrolysis degree disappeared [27]. However, most current in vitro digestibility testing methods do not take these brush border peptidases into account [28,29,30].

The proteins, peptides and even free amino acids that have not been digested and absorbed in the small intestine, eventually end up in the large intestine where they will be fermented by the gut microbiota. Common protein conversion reactions that occur in the large intestine are deamination and decarboxylation, eventually leading to the formation of short chain fatty acids and amines [31]. These reaction products on their turn will trigger a lot of different biological reactions, such as inflammation modulation and signal transduction [31]. Besides the enzymes that are listed above, the pH conditions also changing throughout the gastrointestinal tract will play a key role in protein digestion. Many proteins will lose their structure under acid conditions in the stomach, aiding protein hydrolysis [32].

## 4. Protein Digestibility

The digestibility of proteins is dependent on factors that may both be internal and external to the protein. Internal factors include protein amino acid profile, and protein folding and crosslinking. External factors include pH, temperature and ionic strength conditions, the presence of secondary molecules such as emulsifiers and antinutritional factors. Food processing has a substantial effect on these factors and, hence, protein digestibility. In what follows the internal and external factors contributing to differences in protein digestibility, and the effect of processing on cereal protein digestibility will be highlighted. In addition, both internal and external factors may be influenced by growing conditions (e.g., drought and heat stress) during plant development [33]. However, the preharvest parameters affecting plant protein digestibility fall outside the scope of this manuscript.

### 4.1. Internal Factors

Peptidases often display high specificity to hydrolyse peptide bonds that are neighbouring a specific type of amino acid. Amino acid profiles of proteins, hence, dictate the susceptibility of the protein for hydrolysis by specific peptidases. In addition, these amino acids and the peptide bonds need to be readily accessible for the peptidases. Proline-rich stretches on protein sequences typically reduce the flexibility of the protein chain and are known for their high resistance against peptidase hydrolysis. Gluten proteins, for example, are characterized by high proline levels, which is one of the reasons for its limited digestibility. Tight protein folding or protein aggregation generally restrict access to the peptide chain, and, hence, slow down hydrolysis. Factors affecting protein solubility also influence protein digestibility. Martinez-Velasco and colleagues [34], while studying the digestibility of proteins in maize tortillas during storage, described a negative correlation between protein digestibility and the level of secondary beta-conformations (i.e., beta-sheet and beta-turn conformations) [34]. The limited digestibility of these secondary structures was ascribed to their high hydrophobicity. Both beta-sheet and beta-turn structures were shown to play a crucial role in the formation of a viscoelastic wheat flour dough [35]. At a higher structural level, crosslinking within and between single proteins drastically affects protein digestibility. Geiger and Harris [36] studied the digestibility of wool protein by pepsin. Wool protein is characterised by a dense network structure of peptide chains that are joined through disulfide crosslinks. The reduction of these disulfide bridges, followed by subsequent (partial) reoxidation, showed that the digestibility of those samples with low crosslinking degree was much higher than those proteins that were crosslinked to a greater extent. Upon breadmaking, disulifide bond formation plays a crucial role to dough viscoelasticity and gas retention capacity and final bread quality [37,38].

### 4.2. External Factors

External factors that reduce protein digestibility are the presence of antinutritional factors and physical entrapment in e.g., cellular structures that shield the proteins from the peptidases.

The most well-described antinutritional factors found in plants (and (pseudo)cereals) that limit protein digestibility are protease inhibitors (e.g., trypsin and chymotrypsin inhibitors), tannins and phytates [8,39,40]. Haemagglutinins/lectins in legumes and cereals, glucosinolates in mustard and canola protein products, gossypol in cottonseed protein products, saponins in legumes, oats and tea, and uricogenic nucleic acid bases in yeast protein products are a few other examples [8,41]. Lectins, i.e., noncatalytic sugar-binding proteins, interfere with protein hydrolysis and are themselves known for their high resistance to proteolysis and stability over a wide pH range [41,42]. They can, hence, be found in both fresh and processed food we digest on a daily basis. Although lectins have been traditionally associated with food poisoning, the view of approaching lectins as bioactive proteins in food and feed has recently been gaining traction [42].

Peptidase inhibitors such as trypsin and chymotrypsin inhibitors are also proteins but are much more susceptible to temperature treatments and the hydrolytic conditions in the gastrointestinal tract [40]. (Chymo)trypsin inhibitors will reduce the protein digestibility by inactivating (chymo-)trypsin, two of the most important peptidases in the gastrointestinal tract. However, in the case of trypsin inhibitors, a simple heat treatment is often sufficient to inactivate these antinutritional factors. Trypsin inhibitors are proteinaceous in nature, which means that they can relatively easily be inactivated by heat processing, infrared radiation, boiling, flaking etc. However, Clemente and colleagues [43] found that a substantial fraction of the trypsin inhibitor activity was heat-resistant.

Heat-stable antinutritional factors, conversely, are e.g., tannins [4] and phytates [41,44]. Tannins are naturally occurring, water-soluble polyphenols that form complexes and precipitate with proteins in aqueous environments. By interacting with proteins (peptidases and protein substrates) and minerals, tannins reduce the hydrolytic activity in food [41]. Some of the tannins are easily hydrolysed in acid or alkaline conditions or by enzymes. The condensed tannins, in contrast, are polymerised molecules that are very resistant against hydrolysis [8]. Condensed tannins are the predominant type of tannins found in our food. Tannins are present in high levels in sorghum, millet, various types of beans and peas. As heat treatments do not affect tannin concentration, alternative technological treatments to reduce tannins were developed including dehulling, soaking, addition of chemicals and germination [8,45].

Phytic acid or phytates naturally occur in plants and are known for their metal sequestering activity. In plants phytates function as a mineral source during germination. In the gastrointestinal tract of humans and animals, phytate will sequester essential minerals, making these less bioavailable. Phytate interferes with protein digestibility by competing for mineral cofactors needed by peptidases to be active and in direct interaction with the protein. Phytate content can be reduced by extrusion (high temperature, high shear treatment), while the classical heat treatments do not affect phytate levels [41,44]. In addition, heat treatments inactivate phytases, enzymes known to hydrolyse phytate. In cereals such as rice, wheat and millet, phytate is predominantly found in the outer kernel layers that are typically removed during the milling process. In corn, conversely, phytate is found in the endosperm [46].

Dietary fibre has also been associated with hampered protein hydrolysis. However, the effect of dietary fiber may be a purely physical one, i.e., by increasing the viscosity of the gastrointestinal tract content, hydrolytic enzymes may not as quickly diffuse to and gain access to their substrates [47].

For a more comprehensive review on antinutritional factors that affect peptidase activity, the readers are kindly referred to the excellent review by Gilani and colleagues [8].

### 4.3. Effect of Processing

In general, food processing is meant to increase the nutritional value of food products, by making the biopolymers more readily available for digestive processes [41]. In plant-based food products the gelatinization of starch, disruption of cell walls and the inactivation of toxic and antinutritional factors, need to be achieved to ensure proper energy and nutrient extraction.

In terms of protein digestibility, very early studies indicated that raw egg white is highly indigestible and causes serious gastrointestinal discomfort upon consumption [48]. Upon processing (in this case heating), the digestibility of egg white proteins considerably increased. Early studies pointed to an anti-tryptic factor or special chemical constitution of the proteins, hampering protein digestibility. Of all the different egg white protein constituents present, the albumin fraction was identified as the highly indigestible component. Raw yolk, in contrast, is well-digested and utilized and if gastrointestinal discomfort arises upon consumption of raw yolk, it is often ascribed to the high fat content of this fraction [48]. Later studies indeed confirmed that the true ileal digestibility of cooked and raw egg proteins was very different, i.e., about 90% vs. 50%, respectively [49,50]. In vitro testing with specific peptidases indicated that ovalbumin in raw egg white was only slightly or even not digested, while heat coagulated egg white proteins were much more susceptible to digestion by pepsin [50].

#### 4.3.1. Particle Size Reduction and Physical Size Separation

Cereals and pseudocereals often undergo a size reduction or milling process before being used as ingredients in food production. During this milling or grinding process, cellular structures are sheared open and the protein matrix’s exposure to the environment (and to hydrolytic enzymes) is enlarged. Pure physical size reduction processes, hence, often enhance protein digestibility.

Air classification of cereal fractions is a cheap and clean technique to obtain fractions with different physical properties and chemical composition. These fractions are typically also different in terms of functionality and nutritional quality. Air classification of bran fractions e.g., results in at least some fractions with a considerably higher protein content. One of these higher protein fractions, isolated by Ranhotra and colleagues [51], that had a higher protein content also had a better amino acid profile as a higher amount of the first limiting essential amino acid lysine was measured in this fraction. Furthermore, the apparent protein digestibility of this fraction seemed to be much higher [51].

#### 4.3.2. Heat and Pressure Treatments

The digestibility of proteins generally benefits from thermal denaturation [32]. Depending on the severity of the treatment and the protein type, proteins may either lose their tightly folded structure, leading to higher accessibility of the peptide chain for hydrolytic enzymes, or aggregate. An example of increased digestibility through heat treatment occurs in bean processing. Beans often undergo a soaking and cooking process, processes that Barampama and Simard [52] found improved protein digestibility.

However, the unfolding of proteins may eventually result in (dense) aggregate formation, impairing protein digestibility [53]. Indeed, the formation of, for example, disulfide bonds between sorghum proteins during heat treatments leads to a lower protein digestibility [54]. Research also indicated that the loss of digestibility under in vitro conditions was triggered by hydrophobic interactions among proteins [54]. Heat treatments, hence, have conflicting effects on protein digestibility. While heat inactivation of trypsin inhibitors should positively affect the digestibility scores of proteins, protein denaturation followed by aggregation, triggered by the heat treatment, reduces the protein digestibility [8]. Intense heat treatments may eventually even lead to deterioration or degradation of the amino acid residues [32].

Additionally, the pH, ionic strength and overall product composition will determine the effect of heat treatments on protein configuration and digestibility. Heat treatments under the ‘right’ conditions may promote racemization, Maillard reactions, or the formation of disulfide bridges and other covalent bonds such as lysinoalanine (LAL, especially promoted under alkaline conditions) and isopeptide bonds [8,32,55]. Alkaline/heat treatments induce the formation of D-amino acids in proteins (racemization), that drastically reduce the protein quality (evaluated by monitoring rat growth) [8]. These D-amino acids may be more susceptible to certain degradation reactions than their L-amino acid counterparts, but also hamper protein hydrolysis and, hence, affect the bioavailability of essential amino acids. Pasta products enriched with legume or wheat gluten proteins and dried at different temperatures showed that drying at high temperatures can lead to a decreased amino acid bioavailablity. Structural protein modifications, including Maillard reactions are at the basis of this decreased bioavailability [56]. Apart from disulfide bond formation, heat processing of protein-rich food can also trigger the formation of covalent isopeptide bonds between lysine and glutamic or aspartic acid. Although the reactions lead to a net decrease of the lysine content, the isopeptide bonds seem to be readily hydrolysable. Isopeptide bond formation, hence, likely does not affect the bioavailability of lysine. However, excessive crosslinking through isopeptide bonds, may eventually reduce the accessibility of the protein chain to digestive enzymes [32]. Another type of covalent bonds that are formed under alkaline conditions are LAL bonds, formed in cereal products such as hard pretzels [32,57]. The quasi protective effect of isopeptide formation on lysine bioavailability, however, is an exception. Thermal treatments often trigger derivatization reactions with the ε-amine group on the side chain of the lysine. This makes lysine unavailable as essential amino acid. Examples of molecules with which the amine group can react are reducing sugars, oxidised polyphenols, and oxidised lipids. The available lysine content after processing can be measured using 1-fluoro-2,3-dinitrobenzene [4].

Alkaline treatments in cereal processing are not restricted to pretzelf formation. During corn tortilla production, the alkaline (heat) treatment of the corn, also referred to as nixtamalization, has also been shown to reduce protein digestibility [34].

High hydrostatic pressure treatment is an emerging technology that has lately received a lot of interest as an alternative to heat pasteurization. High pressure pretreatment of cereal flour samples prior to using these flours in baking barely reduced the protein digestibility of the resulting bread samples. A possible explanation for this slight decrease (if any) was the formation of a protein network or intra-/intermolecular disulfide bridges [58]. Pressure treatments induce protein denaturation, but the severity of the denaturation is highly dependent on the processing parameters [59]. In addition, high pressure cooking also leads to the destruction of antinutrients such as phytates, tannins and trypsin inhibitors. Studies describing both a reduction and increase of the protein digestibility can, hence, be found. While high pressure cooking of rice e.g., reduced the protein digestibility more than regular cooking [60], high pressure cooking has also been reported to improve the in vitro protein digestibility of cowpeas [61]. Moreover, high pressure treatments have been proven to avoid or revert the digestibility decreasing effect of cooking on sorghum proteins [62].

#### 4.3.3. Extrusion and Explosion Puffing

Extrusion is a combined thermo-mechanical treatment that alters the chemical, physical and nutritional profile of food products and is a common technique to produce cereal-based snack and breakfast cereal products. Besides the use of extrusion technology to produce cereal-based snack foods, it can also be used to alter flour properties. Extrusion has been shown to increase the digestibility of nutrients quite significantly [28,63]. Molecular scission and the inactivation of anti-nutritional factors through a physical/chemical breakdown have been well-described [41,63]. For cereal bran fractions, for example, extrusion has been used to drastically reduce phytate, polyphenol and trypsin inhibitor levels/activity [41]. Dahlin and Lorenz [64] studied the in vitro digestibility (using trypsin) of extruded wholegrain flours of seven different grains, i.e., rye, winter wheat, quinoa, corn, millet and a low tannin and tannin containing sorghum. Their results showed that protein digestibility of all seven wholegrain flours can be increased by careful selection of the extrusion process conditions [64]. A similar increase in protein digestibility was noted by Omosebi and colleagues [63] after extrusion cooking of an infant formula consisting of protein maize, soybean protein concentrate and cassava starch.

Explosion puffing is a technique in which grains are heated for a short time in an expansion chamber at elevated pressures. After a predetermined time, the chamber is opened and due to the sudden pressure drop, the water in the grains instantaneously evaporates, leading to a drastic expansion of the grains. Huang and colleagues [29] found that explosion puffing improved the protein digestibility of the puffed grains (barley, rice and wheat). They, however, did not observe this improved protein digestibility for millet. The improved digestibility was explained by the more open structure of the puffed grains, increasing the accessibility of the proteins for peptidase activity [29]. A similar protein digestibility increase was observed by Llopart and Drago [65] after the popping of sorghum. The fragmentation of the cell walls in the kernel endosperm was here also discussed as the main cause of the improved digestibility. Conversely, the protein digestibility of sorghum was not affected by popping in a study conducted by Parker and colleagues [66]. It seems that varietal differences and processing conditions play a major role in protein digestibility.

#### 4.3.4. Fermentation and Germination

During germination and fermentation, a myriad of hydrolytic enzymes are released or synthesised that will degrade antinutritional factors (e.g., phytases) or hydrolyse the biopolymers (e.g., proteins) [67]. The digestibility of millet proteins can be increased by simple processing steps such as decortication, germination, fermentation and parboiling [54]. Anti-nutrient levels are decreased during processing and proteins are degraded, while protein extractability is increased [54]. The presence of reducing agents has also been shown to increase protein digestibility by its action on disulfide crosslinks. Additionally, the protein digestibility in a variety of seeds (bread nut, cashew nut and fluted pumpkin) could be improved by boiling and fermentation. In these seeds, tannins, phytic acid and trypsin inhibitors are present that will reduce protein digestibility. Upon boiling, the tannin content was substantially reduced, while fermentation proved to be the most effective processing method to reduce phytic acid and trypsin inhibitor activity [68]. A similar effect on protein digestibility was found by Ogodo and colleagues [69] on fermented sorghum flour. These researchers speculated that hydrolytic enzymes may have converted the highly insoluble storage proteins into more simple and soluble products. Furthermore, the pH drop during fermentation may well favour the enzyme activity of peptidases and increase protein solubility [69].

#### 4.3.5. Protein Hydrolysis

In order to increase the functionality of proteins (the emulsifying, foaming and/or gel forming properties), proteins are often mildly hydrolysed into peptides that have still a relatively high molecular weight [70]. In the specific case of milk proteins, hydrolysis did increase the in vitro protein digestibility [71]. A similar effect was seen on plant and cereal proteins. Koopman and colleagues [72] demonstrated that digestion is accelerated on protein hydrolysates as compared to the intact protein. Also, the absorption from the intestine is accelerated, promoting a quicker incorporation of these amino acids in skeletal muscle protein [72].

#### 4.3.6. Breadmaking Process

Breadmaking is a complex procedure that involves hydration, fermentation and heating steps, hence, combining some of the above processing steps. Wu and colleagues [73] investigated the protein digestibility changes during breadmaking using gluten-containing and gluten-free flours. Protein digestibility was shown to increase during fermentation/proofing, while it decreased again during baking. Furthermore, the same researchers [73] found that the protein digestion rates of the flours were inversely correlated with the total polyphenol and dietary fibre content. Polyphenols are believed to bind to recognition sites of digestive enzymes and as such, hamper the hydrolysis reaction. In addition, protein crosslinking through polyphenols could occur, further limiting protein digestibility [73].

Kostekli and Karabaya [40] studied the levels of trypsin and chymotrypsin inhibitor activities in cereal flours, dough and bread samples. As bread is a staple in our daily diet, information on the levels of these inhibitors and their activities is crucial. During processing, trypsin inhibitor activity decreased upon fermentation and baking. Chymotrypsin inhibitor activity, conversely, increased in wholewheat products during fermentation [40]. In contrast to refined wheat flour, in wholewheat flour, dough and bread, no activity of the trypsin inhibitors was detected. A possible explanation for this is the complex formation between protease inhibitors and bran components, possibly complex polysaccharides, that inactivate the trypsin inhibitors [40].

The suppressed protein digestibility of gluten proteins after baking has been attributed to protein denaturation. Substitution of wheat flour with rice flour has increased protein digestibility in biscuits [74]. Furthermore, in terms of improving the nutritional profile of baked products, diversification in the used cereals can come up to the suboptimal amino acid composition of wheat [75,76].

## 5. Indigestible Proteins

Some specific domains in dietary proteins are very stable against digestion and were even suggested to be able to cross the gut mucosal barrier. These peptides have been hypothesized to be either beneficial to human health (e.g., bioactive peptides) or elicit an immune response (e.g., food allergy) [77,78,79].

Storage proteins of (pseudo)cereals have a lower digestibility than animal proteins. Buckwheat proteins e.g., have a low availability to gastrointestinal absorption. The poor availability of buckwheat proteins is caused by high levels of protease inhibitors and tannins and the low susceptibility of the proteins, especially the albumin fraction, to proteolytic activity [39]. Additionally, millet is known for its lower starch and protein digestibility rate relative to other cereals [30]. Another cereal, sorghum, predominantly grown in semi-arid tropical areas, has a very poor protein digestibility after wet cooking. The poor protein digestibility in both sorghum and millet stems from the dense internal grain structure, the presence of polyphenols and phytic acid, the formation of disulfide and non-disulfide crosslinks, protein hydrophobicity and changes in secondary structure that were induced during wet cooking [9]. Protein crosslinking of the kafirin molecules in sorghum and the formation of hydrophobic aggregates of panicin in millet are probably the major factors in reducing protein digestibility [6,9]. Teff proteins are relatively well-digestible, but their digestbility can be further increased by cooking teff into injera [80].

Wheat proteins have been associated with a number of dietary disorders. The best well-known disorder is celiac disease, a disorder that develops in genetically susceptible individuals after ingesting gluten-containing cereals. Wheat gliadins, and to a lesser extent low molecular weight glutenins, carry immunogenic peptides [81]. A variety of these celiac-disease-initiating peptides of α-gliadin have been identified. Examples of some of these immunogenic epitopes are glia-α9 (PFPQPQLPY) and glia-α20 (FRPQQPUPQ) [82]. The unusual amino acid composition (high proline and glutamine contents) in gluten proteins prevents the complete digestion of these proteins in the gastrointestinal tract. While for most people the peptides do not cause any problems, an estimated 1% of the world population suffers from celiac disease [83] and in these individuals, these peptides trigger a cascade of auto-immune reactions that lead to severe intestinal damage. Several researchers have been trying to develop solutions for people suffering from celiac disease. One of these investigated solutions involves the pretreatment of the gluten protein with peptidase mixtures (e.g., papaya non-specific endopeptidase and three microbial peptidases (*Aspergillus oryzae* leucine aminopeptidase, *Aspergillus melleus* endopeptidase with activity against hydrophibic amino acid residues and *Penicillium citrinum* deutorlysin)) [84]. These peptidases are able to digest the above proline-rich peptides and, hence lower the concentration of the immunogenic peptides. Other strategies include the development of wheat varieties that do not trigger these gastrointestinal responses [81] and targeted processing of the cereals. One of such novel cereals is tritordeum, a hybrid of durum wheat and wild barley [85]. Tritordeum was shown to have lower numbers of immunogenic epitopes than regular wheat. This novel cereal is suitable to include in diets for people that want to reduce their gluten intake, but not for people suffering from celiac disease as there are still gluten immunogenic peptides produced upon digestion [85]. Processing has a big effect on the physicochemical properties of gluten, and will, hence, affect the digestive stability, and, hence, the antigenic potential of the protein [86]. Rahaman and colleagues [86] found that shear by itself does not affect protein digestibility, while pH and temperature substantially affect gluten digestibility and the antigenic characteristics of the hydrolysates that are formed. At pH 3, gluten undergoes acidic deamidation that will lead to a better hydrolysis of the proteins, generating smaller peptide fractions with a lower antigenicity [86,87]. When heating proteins, proteins are aggregating, increasing the resistance of the proteins against digestion [86]

## 6. Outlook

Proteins of cereals and pseudocereals are not considered of high quality due to their suboptimal amino acid profile and limited protein digestibility. Protein digestibility is often affected by a combination of different factors that are specific for the protein under study and/or its environment. Protein digestibility can, hence, be modulated by selective food processing. However, the effect of processing on protein digestibility is not that straightforward and tight control of the processing conditions is needed. Furthermore, the need for increasing protein digestibility in our common Western world diet is questionable as protein consumption is usually not a limiting factor in the Western diet. One exception and point of attention in this context, however, is the elder segment of the Western population that requires adequate protein intake through (and even protein enrichment of) their diet in order to maintain muscle mass and function [21,22,23,24]. A concern when consuming cereal proteins is the suboptimal amino acid ratios that do not match the ratios a human should consume to support growth and a healthy metabolism. A more diversified consumption of cereals and pseudocereals, surpassing the traditional wheat- and corn-dominated diet, could partially come up to these suboptimal ratios.
